# Disability and Health Data System Updated

**Published:** 2013-06-28

CDC has updated its Disability and Health Data System (DHDS), a web-based data tool providing state-level data on the health of adults with disabilities. Users can now view data in state profiles, in addition to maps and data tables. State profiles allow users to view several pieces of health information simultaneously by topic area for their state. Additionally, 2011 Behavioral Risk Factor Surveillance System (BRFSS) data have been added to DHDS and are included in maps, data tables, and state profiles. New analyses were added for the 2011 data: health indicators stratified by age, sex, and race/ethnicity, and p-values for health disparities. Indicator labels also were revised to make it easier to interpret the information being viewed.

DHDS is intended to raise awareness of health disparities experienced by adults with disabilities and to inform program and policy initiatives aimed at improving the health of adults with disabilities. Recent data on the health of adults with disabilities in specific states and health program areas are available through DHDS at http://dhds.cdc.gov. Questions about the system can be sent to dhds@cdc.gov.

